# Targeting oncogenic vulnerabilities in triple negative breast cancer: biological bases and ongoing clinical studies

**DOI:** 10.18632/oncotarget.14731

**Published:** 2017-01-18

**Authors:** Alberto Ocana, Atanasio Pandiella

**Affiliations:** ^1^ Unidad de Investigación Traslacional, Hospital Universitario de Albacete, Universidad de Castilla La Mancha, Albacete, Spain; ^2^ Instituto de Biología Molecular y Celular del Cáncer and CIBERONC. CSIC-Universidad de Salamanca, Salamanca, Spain

**Keywords:** triple negative breast cancer, breast cancer, kinases, novel therapies

## Abstract

Triple negative breast cancer (TNBC) is still an incurable disease despite the great scientific effort performed during the last years. The huge heterogeneity of this disease has motivated the evaluation of a great number of therapies against different molecular alterations. In this article, we review the biological bases of this entity and how the known molecular evidence supports the current preclinical and clinical development of new therapies. Special attention will be given to ongoing clinical studies and potential options for future drug combinations.

## INTRODUCTION

Triple negative breast cancer (TNBC) is a clinicopathological entity which includes breast tumors that do not express immunohistochemically detectable estrogen, progesterone and HER2 receptors [1]. They account for around 15% of all breast cancers, being more frequently expressed in young women and those from African and Hispanic descendants [1–3]. Genomic studies have revealed the heterogeneous nature of TNBC [4–6]. Indeed, this entity has been classified by gene expression analyses in several subgroups, including two basal-like (BL1, BL2), an immunomodulatory (IM), a mesenchymal (M), a mesenchymal stem-like (MSL) and a luminal/androgen receptor (LAR) subtype [5]. A similar study but using a smaller dataset described four groups that mimic the previous reported subtypes: luminal/androgen receptor (LAR), mesenchymal (MES), basal like/immune-suppressed (BLIS) and basal like/immune activated (BLIA) [7].

Although heterogeneous, most TNBC share common clinical features such as poor long term prognosis or a specific pattern of relapse, mainly during the first five years after diagnosis [8]. In addition, some data suggests that the IM and BLIA subgroups are associated with an enrichment of lymphocytes and are those with better outcome [5, 7]. These findings are in line with recent studies suggesting that TNBC with tumor-infiltrating lymphocytes (TILs) have better prognosis [9, 10].

Due to the lack of druggable known targets most patients with TNBC are treated with chemotherapy [11]. Chemotherapy regimens have clinical activity in some TNBC patients. The group of patients that obtain a pathological complete response (pCR) after neoadjuvant chemotherapy, have a better clinical outcome than those that do not achieve such response [12]. However, in the metastatic setting chemotherapy shows limited efficacy and most patients soon progress to these agents.

The poor clinical prognosis, the limited long term efficacy of chemotherapy and the absence of targeted therapies support research to identify new targets and develop novel therapies against this cancer. In this review we will discuss novel biological findings that are therapeutically exploitable as well as combinations of agents to efficiently augment and optimize existing therapies.

## NOVEL THERAPEUTIC POSSIBILITIES IN TNBC

**Figure 1:**
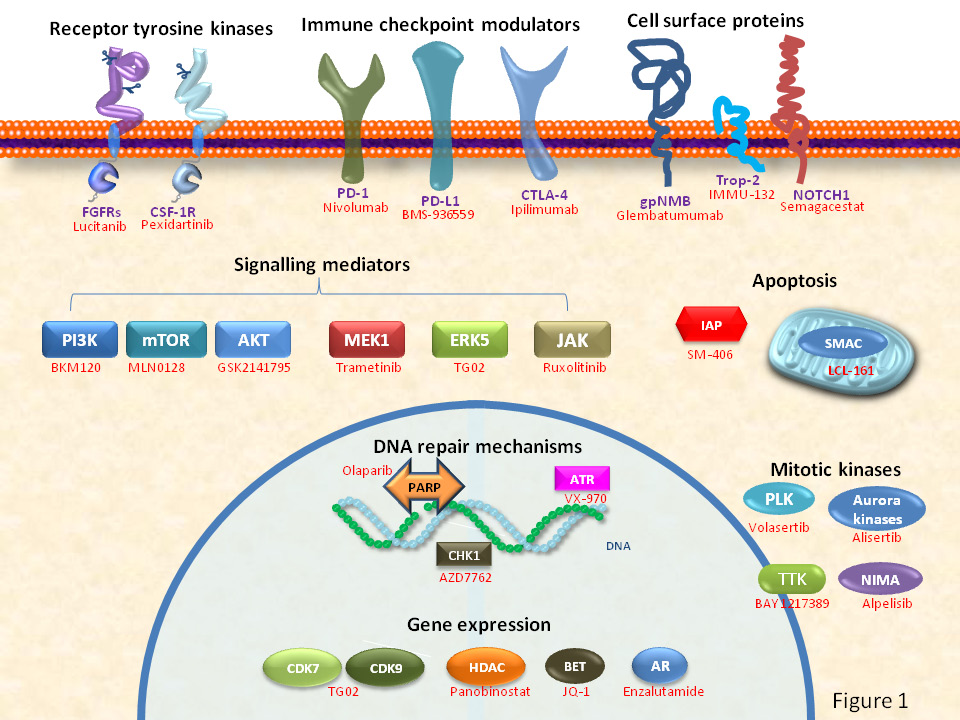
Schematic representation of cellular functions and key components used as potential drug targets for the therapy of TNBC Examples of drugs already approved or under clinical development are shown in red.

Molecular and functional studies have unraveled cellular functions that are important in the generation/progression of TNBC. On the bases of these studies, agents targeting distinct components of those functions have been developed. Figure 1 provides a schematic view of key components of such cellular functions for whom therapeutic agents have been developed. Some of them, already approved or under clinical evaluation, are shown.

### DNA repair mechanisms

**Figure 2:**
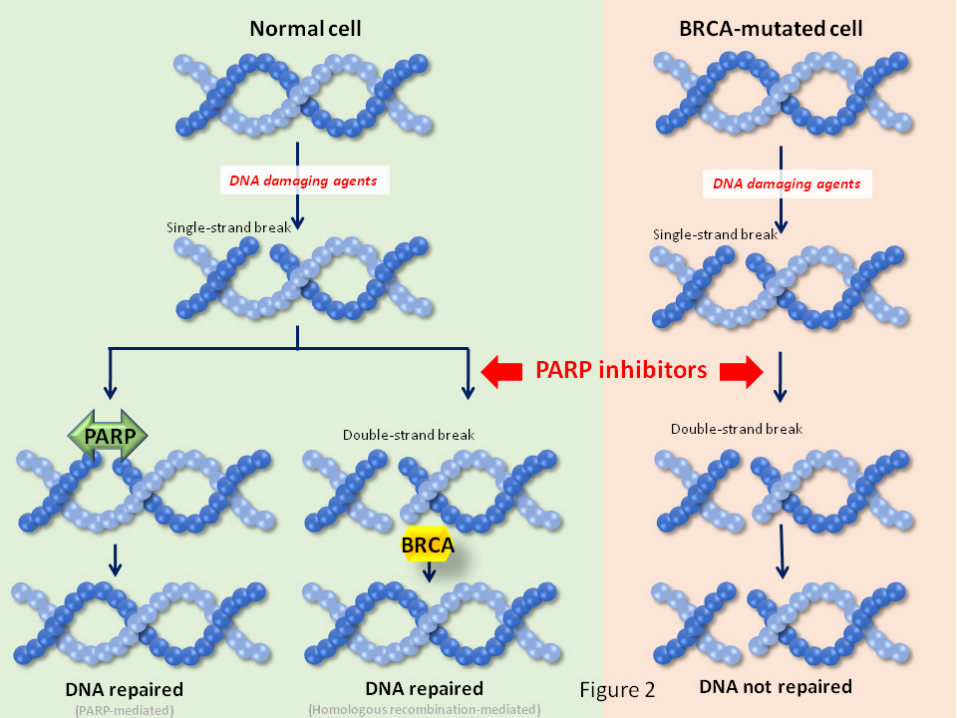
Synthetic lethality interactions for PARP inhibitors In synthetic lethality two different pathways participate to sustain a cellular function. In the case shown in the figure, single strand DNA repair is accomplished by the action of PARP. In case the lesion progresses to a double strand break, it can be repaired by the action of BRCA1. In case one of the pathways is impaired (e. gr. by loss of function of BRCA1), the cellular function is still supported by the action of the other pathway. An alteration in a base, or a single strand break is usually repaired by the base excision repair mechanism, in which PARP proteins play an essential role. If inhibitors of PARP are used, then the single strand alteration may evolve to double strand breaks. The repair of lesions depends on the integrity of BRCA proteins. In patients whose tumors are defective in BRCA activity, the PARP inhibitors create DNA double strand breaks that are not repaired, leading to cell death.

In TNBC alterations of the DNA repair machinery seem to be of significance, as there is an increased presence of somatic or acquired mutations in DNA repair genes, mainly BRCA1 or BRCA2 genes. Those genes code for key tumor-suppressor proteins that are important components of the homologous recombination DNA repair mechanism [13, 14] (Figure 2). In this context, chemotherapies that affect DNA like platinum compounds, and agents that act by inhibiting the PolyADP-Ribose Polymerase (PARP) protein have been evaluated in this subtype of breast cancer. Platinum chemotherapy causes DNA damage through induction of adducts or DNA crosslinking. These lesions are normally repaired through base excision repair mechanisms in which PARP1 plays an essential role. Platinum compounds have moderate activity in unselected TNBC tumors but are more active than taxanes particularly in tumors harboring BRCA mutations [15, 16].

Given the fact that in BRCA1/2 mutated tumors PARP inhibitors produce a synthetic lethality interaction [17, 18] (see Figure 2), these compounds are currently in clinical development in this indication. Recently, olaparib, an agent that targets several PARP isoforms including PARP1, PARP2 and PARP3, has been approved for the treatment of ovarian cancer in patients harboring deleterious mutations of the BRCA1/2 genes [19, 20]. In TNBC several phase II trials have evaluated various PARP inhibitors. Olaparib showed clinical activity when patients were selected for BRCA1 mutations, with response rates from 13-50% depending on the number of prior chemotherapies or the previous exposure to platinum-compounds [20, 21]. Of note, in unselected patients efficacy was very limited [22]. Velaparib is another inhibitor that targets PARP1 and PARP2. In a phase II trial clear signs of activity were observed with velaparib in combination with temozolomide in BRCA1 mutated patients [23]. Ongoing phase III studies with PARP inhibitors are shown in Table 1.

**Figure 3:**
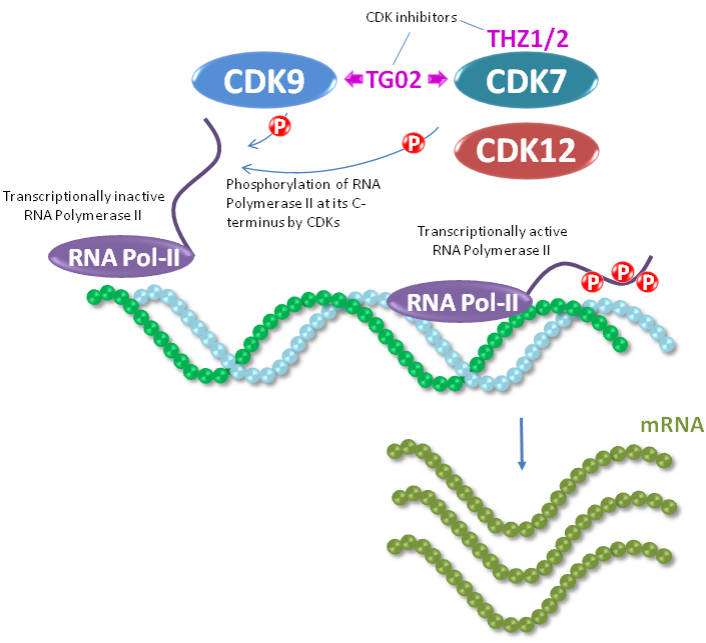
CDK7 and CDK9 act as regulators of RNA polymerase II, an enzyme critical in mRNA production which is required for protein synthesis RNA polymerase II is recruited to gene promoters by transcription factors, and is phosphorylated in its C-terminal domain heptad repeats. At these heptad repeats, CDK7 phosphorylates RNA polymerase II on serine 5 during initiation of transcription, and CDK9 phosphorylates serine 2 during elongation of the transcripts. The activity of CDK7 and CDK9 can be targeted by the dual inhibitor TG02.

**Table 1:** Summary of ongoing phase III studies in TNBC describing the new compound, indication and the study design

Study number	Indication	New compound	Control Arm	Experimental Arm
NCT02032277	Early Stage Triple Negative Breast Cancer	ABT-888 (PARP inhibitor)	Active Comparator: Arm A Veliparib + carboplatin + paclitaxel followed by doxorubicin/cyclophosphamide (AC)	Placebo Comparator: Arm B Placebo + carboplatin + paclitaxel followed by AC. Arm C Placebo + placebo + paclitaxel followed by AC.
NCT02555657	Metastatic Triple Negative Breast Cancer	Pembrolizumab (MK-3475) (Anti-PD-1 Monoclonal Antibody)	Experimental: Pembrolizumab Participants receive pembrolizumab 200 mg intravenously (IV) every 3 weeks (Q3W) for up to 35 administrations	Active Comparator: Chemotherapy Participants receive capecitabine, eribulin, gemcitabine, or vinorelbine as therapy of physicians choice in accordance with local regulations and guidelines
NCT02574455	Refractory/Relapsed Triple-Negative Breast Cancer	Sacituzumab Govitecan IMMU-132 (ADC against Trop-2 linked to irinotecan metabolite SN-38)	Experimental: IMMU-132 Sacituzumab govitecan (10 mg/kg on Days 1 and 8 of 21-day cycles)	Active Comparator Treatment of Physician's: Eribulin (1.4 mg/m2 intravenously on Days 1 and 8 of a 21-day cycle). Capecitabine (1250 mg/m2 orally twice day for 2 weeks followed by a 1 week rest period given as a 21-day cycle). Gemcitabine (1250 mg/m2 intravenously on Days 1 and 8 of a 21-day cycle). Vinorelbine (30 mg/m2 weekly; IV injection over 6-10 min)
NCT02425891	Previously Untreated Metastatic Triple Negative Breast Cancer	Atezolizumab (MPDL3280A) (Anti-PD-1 Monoclonal Antibody)	Active Comparator: Atezolizumab plus nab-paclitaxel	Placebo Comparator: Placebo and nab-paclitaxel
NCT00938652	ER-, PR-, and Her2-Negative Metastatic Breast Cancer	BSI-201. Iniparib (PARP inhibitor)	Active Comparator: Arm G/C gemcitabine/carboplatin on Days 1 and 8 of 21-day cycle(s)	Experimental: Arm G/C/I gemcitabine/carboplatin on Days 1 and 8, plus iniparib on Days 1, 4, 8, and 11 of 21-day cycle(s)

Abbreviations: ER, estrogen receptor; PR, progesterone receptor.

Other druggable proteins that are involved in DNA repair include ATR or its effector kinase Chk1 [24]. Their inhibition has shown promising preclinical activity in TNBC that lack the expression of ERCC1 [25]. In this context, agents targeting these proteins are currently in clinical development (see Tables 2 and 3). Finally, agents that bind DNA like novel mithramycin analogs (mithralogs) or trabectedin have also shown activity in preclinical models, but their potential antitumoral activity in the clinical setting requires evaluation [26, 27].

**Table 2:** List of ongoing phase II trials in TNBC as from clinicaltrials.gov, access Jun 1, 2016

Study number	New compound	Study design	Indication
NCT01745367	Triple Negative Breast Cancer (TNBC)	Tivozanib Hydrochloride (AV-951, an oral VEGF receptor tyrosine kinase inhibitor )	Active Comparator: Placebo in combination with paclitaxel Placebo orally once daily on a 3 weeks on/1 week off schedule with 90 mg/m2 of paclitaxel administered intravenously 3 weeks on (Day 1, Day 8 and Day 15)/1 week off (4 weeks = 1 Cycle). Experimental: Tivozanib Hydrochloride in combination with paclitaxel 1.5 mg tivozanib hydrochloride orally once daily on a 3 weeks on/1 week off schedule with 90 mg/m2 of paclitaxel administered intravenously 3 weeks on (Day 1, Day 8 and Day 15)/1 week off (4 weeks = 1 Cycle).
NCT02393794	Triple Negative Breast Cancer	Romidepsin (histone deacetylase inhibitor)	Experimental: Romidepsin (8mg/m2) + Cisplatin (75mg/m2) Romidepsin 8mg/m2 IV on days 2 & 9 of each 21 day cycle Cisplatin 75mg/m2 IV on day 1 of each 21 day cycle Experimental: Romidepsin (10mg/m2) + Cisplatin (75mg/m2) Romidepsin 10mg/m2 IV on days 2 & 9 of each 21 day cycle Cisplatin 75mg/m2 IV on day 1 of each 21 day cycle Experimental: Romidepsin (12mg/m2) + Cisplatin (75mg/m2) Romidepsin 12mg/m2 IV on days 2 & 9 of each 21 day cycle Cisplatin 75mg/m2 IV on day 1 of each 21 day cycle Experimental: Romidepsin Dose Expansion Romidepsin maximum tolerated dose (MTD) from Phase I IV on days 2 & 9 of each 21 day cycle Cisplatin 75mg/m2 IV on day 1 of each 21 day cycle
NCT02161679	Triple Negative Breast Cancer	IMMU-132 Antibody drug conjugate	Experimental: IMMU-132 Active Comparator: IMMU-132 plus Carboplatin
NCT01997333	gpNMB Over-Expressing, Triple Negative Breast Cancer (METRIC)	CDX-011 glembatumumab vedotin (antibody-drug conjugate)	Active Comparator: Capecitabine Capecitabine will be administered on Days 1 through 14 of each 21 day cycle. Experimental: Drug: CDX-011 CDX-011 administered as an intravenous infusion on Day 1 of each 21 day cycle.
NCT02593175	Localized Triple-Negative Breast Cancer (TNBC)	Panitumumab (anti-EGFR mAb)	Experimental: Panitumumab + Carboplatin + Paclitaxel One week before Cycle 1 participants receive a single dose of Panitumumab 1000 mg by vein. About 1 week after the first Panitumumab dose, participants have an image-guided core biopsy and/or a fine needle aspiration (FNA) to remove breast tissue. Participants then receive the study drug combination for 4 cycles. Each cycle is 21 days. On Day 1, 8, and 15 of each cycle, participants receive Panitumumab 2.5 mg by vein and Paclitaxel 80 mg/m2 by vein. On Day 1 of each cycle Carboplatin AUC 6 received by vein.
NCT01307891	Metastatic, Triple Negative Breast Cancer	Abraxane ( formulation of paclitaxel)	Experimental: Combination Abraxane and Tigatuzumab Patients will receive Abraxane at 100 mg/m2 × 3 doses on Days 1, 8, and 15 at 28-day intervals in combination with tigatuzumab to be administered as a 10 mg/kg loading dose followed by 5 mg/kg for the first cycle and then every other week on Days 1 and 15 for subsequent cycles. Patients will be evaluated for response every 8 weeks. Patients with disease progression will be taken off the study. Experimental: Abraxane alone Patients will receive Abraxane at 100 mg/m2 weekly X 3 doses on Days 1, 8, and 15 at 28-day intervals. Treatment may continue without interruption in patients with complete response (CR), partial response (PR), or stable disease (SD) until there is progression of the disease or unacceptable toxicity. Patients will have the option to crossover to the combination arm based upon the pre-clinical data.
NCT02402764	Metastatic Triple Negative Breast Cancer	Selinexor (KPT-330) is a first in class SINE™ XPO1 antagonist)	Experimental: Selinexor Treatment
NCT02368691	Androgen Receptor-Positive Triple Negative Breast Cancer (AR+ TNBC)	GTx-024 (selective androgen receptor modulator)	Experimental: GTx-024 GTx-024 capsules, 18 mg PO once-daily for up to 12 months
NCT01617668	Triple Negative Breast Cancer	LCL161 ( is a small molecular antagonist of the inhibitor of apoptosis (IAP))	Experimental: Paclitaxel with LCL161 Active Comparator: Paclitaxel without LCL161
NCT01176669	Triple Negative Breast Cancer	Apatinib (VEGFR2 TK inhibitor).	Drug: Apatinib Apatinib was administratered at 750 mg/d in Phase IIa. The actual average dose intensity delivered was 525 mg/d due to toxicities. So, in Phase IIb, the starting dose of apatinib will be 500mg/d. Two dose reductions will be allowed to 375 and then 250 mg/d.
NCT00813956	Triple Negative Breast Cancer	BSI-201 (Parp inhibitor)	Experimental: standard chemotherapy plus BSI-201
NCT02000882	TNBC BC Brain Met	BKM120 ( PI3K inhibitor)	Experimental: BKM120 plus Capecitabine BKM120 will be administered at a dose of 100 mg orally (PO) daily. Capecitabine will be administered at a dose of 1000 mg/m2 orally (PO) twice a day (rounded down to the nearest 500 mg pill) 14 days on and 7 days off.
NCT00951054	Advanced, Metastatic Triple Negative Breast Cancer	NK012 ( is an SN-38-releasing polymeric micelle)	
NCT02435680	Advanced Triple Negative Breast Cancer (TNBC)	MCS110 ( a monoclonal antibody with potent neutralizing activity against macrophage colony-stimulating factor for the treatment of tumor-induced osteolysis)	Experimental: Arm 1: MCS110+carboplatin+gemcitabine MCS110+carboplatin+gemcitabine Active Comparator: Arm 2: carboplatin+gemcitabine carboplatin+gemcitabine
NCT01964924	Metastatic Triple-Negative Breast Cancer	GSK2141795 (Akt Inhibitor)	Experimental: Treatment (trametinib, Akt inhibitor GSK2141795) PART 1: Patients receive trametinib PO QD on days 1-28. Courses repeat every 28 days in the absence of disease progression or unacceptable toxicity. Patients who experience disease progression continue to Part 2. PART 2: Patients receive trametinib as in Part 1 and also receive Akt inhibitor GSK2141795 PO QD on days 1-28. Courses repeat every 28 days in the absence of disease progression or unacceptable toxicity.
NCT01639248	Previously Treated Locally Advanced + Metastatic TNBC	ENMD-2076 ( Aurora + Angiogenic Kinase Inhibitor)	Experimental: ENMD-2076 Treatment ENMD-2076
NCT02447003	Metastatic Triple-Negative Breast Cancer	Pembrolizumab (MK-3475)	Experimental: Pembrolizumab Participants receive pembrolizumab, 200 mg intravenously (IV) on Day 1 of each 3-week cycle (Q3W) for up to 24 months
NCT02203513	BRCA1/2 Mutation Associated Breast or Ovarian Cancer, Triple Negative Breast Cancer, High Grade Serous Ovarian Cancer, and Metastatic Castrate-Resistant Prostate Cancer	LY2606368 (Chk1/2 Inhibitor)	Experimental: 1 Women with gBRCAm associated breast or ovarian cancer Experimental: 2 HGSOC at low genetic risk Experimental: 3 TNBC at low genetic risk
NCT02162719	Metastatic Triple-Negative Breast Cancer	Ipatasertib (GDC-0068) (Akt inhibitor)	Experimental: Arm 1 Paclitaxel + Ipatasertib Active Comparator: Arm 2 Paclitaxel + Placebo
NCT01045304	Metastatic Triple Negative Breast Cancer	SAR240550 (BSI-201) is a PARP1 inhibitor.	Experimental: Gencitabine + iniparib twice weekly Gemcitabine, 1000 mg/m^2^ IV over 30 minutes and carboplatin, area under the curve (AUC) = 2, IV over 60 minutes, both on Days 1 and 8 of 3-week cycles. Iniparib, 5.6 mg/kg IV over 60 minutes on Days 1, 4, 8 and 11 of 3-week cycles Experimental: Gencitabine + iniparib weekly Gemcitabine, 1000 mg/m^2^ IV over 30 minutes and carboplatin, area under the curve (AUC) = 2, IV over 60 minutes, both on Days 1 and 8 of 3-week cycles. Iniparib, 11.2 mg/kg IV over 60 minutes on Days 1 and 8 of 3-week cycles
NCT01953536	Triple Negative Breast Cancer	Vintafolide (ADC against the folate receptor linked with vinblastine)	Experimental: Vintafolide Participants receive intravenous (IV) vintafolide 2.5 mg on Days 1, 3, 5, 15, 17, and 19 of a 28-day cycle. Experimental: Vintafolide + Paclitaxel Participants receive IV vintafolide 2.5 mg on Days 1, 3, 5, 15, 17, and 19 of one 28-day cycle and receive IV paclitaxel on Days 1, 8, 15, and 22 of a 28-day cycle. Active Comparator: Paclitaxel Participants receive IV paclitaxel on Days 1, 8, 15, and 22 of a 28-day cycle.
NCT01074970	Triple Negative Breast Cancer	Rucaparin (PARP Inhibitor)	Active Comparator: Arm A: Cisplatin Monotherapy Cisplatin 75 mg/m2 IV infusion over 60 minutes, D1 every 21 days for 4 cycles Active Comparator: Arm B: Combination Therapy Rucaparib 24mg C1,30mg C2-4, D1,2,3 every 21 days for 4 cycles Cisplatin 75 mg/m2 IV infusion over 60 minutes, D1 every 21 days for 4 cycles
NCT02301988	Early Stage Triple Negative Breast Cancer	Ipatasertib (GDC-0068) (AKT inhibitor)	Experimental: Arm 1: Paclitaxel + Ipatasertib Placebo Comparator: Arm 2: Paclitaxel + Placebo
NCT01204125	Triple Negative Breast Cancer	Iniparib (SAR2405550 -BSI-201) (Parp inhibitor)	Experimental: SAR240550 twice weekly/ paclitaxel weekly SAR240550 will be administered at the dose of 5.6mg/kg as a 60-min intravenous (IV) infusion. Patients will receive SAR240550 infusions twice weekly (day 1 and day 4; total dose of 11.2mg/kg per week) and paclitaxel weekly as a 60-min IV infusion (day 1; dose of 80mg/m2). Experimental: SAR240550 weekly/ paclitaxel weekly SAR240550 will be administered at the dose of 11.2 mg/kg as a 60-min intravenous (IV) infusion. Patients will receive SAR240550 infusions once weekly (day 1; total dose of 11.2mg/kg per week) and paclitaxel weekly as a 60-min IV infusion (day 1; dose of 80mg/m2). Active Comparator: Paclitaxel alone Paclitaxel will be administered at the dose of 80mg/m2 as a 60-min IV infusion. Patients will receive weekly (day 1) paclitaxel infusions.
NCT02299635	Advanced Breast Cancer With Or Without Notch Alterations	PF-03084014 A selective gamma secretase (GS) inhibitor	Experimental: PF-03084014 PF-03084014 will be administered orally, continuously, twice daily at 150 mg, but the dose can be reduced to 100 mg or 80 mg.
NCT01629615	Triple Negative Metastatic Breast Cancer	BKM120 (PI3K Inhibitor)	Drug: BKM120 BKM120 oral capsules. 100 mg daily in cycles of 28 days, until disease progression
NCT01421472	HER2-negative Breast Cancer	MM-121 (SAR256212) , is an investigational human monoclonal antibody	Experimental: MM-121 (SAR256212) + paclitaxel Active Comparator: Paclitaxel
NCT01698281	Refractory in Triple Negative Breast Cancer	AEZS-108 zoptarelin doxorubicin (an LHRH agonist linked to doxorubicin)	Experimental: AEZS-108 AEZS-108 (267 mg/m2, 2-hour IV infusion every Day 1 of a 21-day (3-week) cycle Active Comparator: Standard single agent cytotoxic chemotherapy commercially available SSCC (doses below the recommended package insert at the discretion of treating oncologist), on a 21-day cycle (although weekly administration is allowed; note: pegylated liposomal doxorubicin will be administered on a 28-day cycle).
NCT02423603	Triple-Negative Advanced or Metastatic Breast Cancer	AZD5363 (serine/threonine AKT/PKB, protein kinase B kinase inhibitor)	Active Comparator: Paclitaxel + AZD5363 Patients receive Paclitaxel on Day 1, Day 8 and Day 15 plus AZD5363/Placebo on Days 2-5, Days 9-12, and Days 16-19. Upon Paclitaxel withdrawal, Patient receive AZD5363/Placebo on Days 2-5, Days 9-12, Days 16-19 and Days 23-27. Placebo Comparator: Paclitaxel + Placebo Patients receive Paclitaxel on Day 1, Day 8 and Day 15. plus AZD5363/Placebo on Days 2-5, Days 9-12, and Days 16-19. Upon Paclitaxel withdrawal, Patient receive AZD5363/Placebo on Days 2-5, Days 9-12, Days 16-19 and Days 23-27.
NCT00540358	Triple Negative Metastatic Breast Cancer	BSI-201 iniparib (Parp Inhibitor)	Active Comparator: Arm G/C Standard chemotherapy with gemcitabine/carboplatin on Days 1 and 8 of 21-day cycle(s) Experimental: Arm G/C/I Standard chemotherapy with gemcitabine/carboplatin on Days 1 and 8, plus iniparib on Days 1, 4, 8, and 11 of 21-day cycle(s)
NCT01818999	Metastatic Breast Cancer	Ixabepilone	Experimental: arm one IXABEPILONE and STEREOTACTIC BODY RADIATION THERAPY (SBRT)
NCT01127763	Breast Cancer	RAD001 (mTOR inhibitor)	Experimental: RAD001+carboplatin Carboplatin (starting dose was initially AUC 6, later decreased to AUC 5, then AUC 4) every 3 weeks as IV infusion and RAD001 as 5 mg pill each day until disease progression or unacceptable toxicity.
NCT00827567	Triple Negative Metastatic Breast Cancer	RAD001 (mTOR inhibitor)	Experimental: RAD 001 RAD001-10 mg by mouth once everyday
NCT01319539	Stage I, Stage II, or Stage III Breast Cancer	MK2206 (AKT inhibitor)	Experimental: Treatment (Akt inhibitor MK2206) Patients receive Akt inhibitor MK2206 PO on days -9 and -2, and undergo segmental resection or total mastectomy on day 0.
NCT01234402	Previously Treated Breast Cancer Patients	IMC-18F1 (Anti-VEGFR)	Experimental: Ramucirumab DP + Capecitabine Cycles repeat until disease progression, the development of unacceptable toxicity, noncompliance, or withdrawal of consent by the patient. Experimental: IMC-18F1 + Capecitabine Cycles repeat until disease progression, the development of unacceptable toxicity, noncompliance, or withdrawal of consent by the patient. Active Comparator: Capecitabine Crossover Study
NCT01234402	Previously Treated Breast Cancer Patients	Ramucirumab (Anti-VEGFR2)	Experimental: Ramucirumab DP + Capecitabine Cycles repeat until disease progression, the development of unacceptable toxicity, noncompliance, or withdrawal of consent by the patient. Experimental: IMC-18F1 + Capecitabine Cycles repeat until disease progression, the development of unacceptable toxicity, noncompliance, or withdrawal of consent by the patient. Active Comparator: Capecitabine Crossover Study

Abbreviations: VEGF, Vascular Endothelial Growth Factor; TK, tyrosine kinase.

**Table 3:** List of ongoing phase I studies in TNBC, as from clinicaltrials.gov, access Jun 1, 2016

Study number	Indication	New drug	Treatment in combination
NCT02622074	Triple Negative Breast Cancer	Pembrolizumab (MK-3475) A humanized monoclonal immunoglobulin (Ig) G4 antibody directed against human cell surface receptor PD-1	Nab-paclitaxel Anthracycline Cyclophosphamide Carboplatin
NCT00707707	Metastatic Triple Negative Breast Cancer	AZD2281/Olaparib (inhibitor of PARP1/2)	Paclitaxel
NCT01884285	Triple Negative Breast Cancer Advanced Castrate-resistant Prostate Cancer (CRPC); Squamous Non-Small Cell Lung Cancer (sqNSCLC);	AZD8186 (inhibitor of PI3Kβ and PI3Kδ)	
NCT01238952	Solid Tumors With Dose Expansion in Triple Negative Breast Cancer	NK012 (SN-38-releasing polymeric micelle)	Carboplatin
NCT01624441	Metastatic Triple-Negative Breast Cancer	Dinaciclib ( CDK inhibitor)	Epirubicin Hydrochloride
NCT02474173	Advanced Triple Negative Breast Cancer	AT13387 (HSP90 Inhibitor)	Paclitaxel
NCT01939418	Metastatic Triple Negative Breast Cancer	RAD001 (mTOR inhibitor)	
NCT01238133	Metastatic Triple Negative Breast Cancer	RO4929097 (Gamma-Secretase/Notch Signalling Pathway Inhibitor)	
NCT01618136	Advanced Solid Tumors or With B-cell Malignancies oradvanced solid tumors	E7449 Poly(ADP-Ribose) Polymerase (PARP) Inhibitor	Temozolomide (TMZ) or With Carboplatin and Paclitaxel
NCT02627430	Metastatic Advanced Solid Tumor or Recurrent Ovarian, Fallopian Tube, Primary Peritoneal, or Triple Negative Breast Cancer	Talazoparib AT13387 (HSP90 Inhibitor)	
NCT02583542	Advanced Cancers (TORCMEK)	AZD2014 mTOR serine/threonine kinase (TORC1/2) inhibitor	Selumetinib
NCT01623349	Recurrent Triple Negative Breast Cancer or High Grade Serous Ovarian Cancer	BKM120 (oral PI3kinase Inhibitor)	Olaparib
NCT01623349	Recurrent Triple Negative Breast Cancer or High Grade Serous Ovarian Cancer	BYL719 (Oral PI3kinase Inhibitor)	Olaparib
NCT01445418	Ovarian Cancer	AZD2281 (PARP1/2 inhibitor)	Carboplatin
NCT02071862	Solid tumors	CB-839 (Glutaminase Inhibitor)	
NCT02259114	Advanced Solid Tumors	OTX015, a Small Molecule Inhibitor of the Bromodomain and Extra-Terminal (BET) Proteins	
NCT01698281	Refractory in Triple Negative Breast Cancer	AEZS-108 zoptarelin doxorubicin an LHRH agonist linked to doxorubicin in women with platinum refractory	
NCT01876251	Advanced Breast Cancer	PF-03084014 (gamma secretase inhibitor)	Docetaxel
NCT02632448	Advanced or Metastatic Cancer	LY2880070	Gemcitabine
NCT02476955	Breast Cancer Triple Negative	ARQ 092 (Akt Inhibitor)	Carboplatin Plus Paclitaxel
NCT02027376	Triple Negative (TN) Advanced Breast Cancer (ABC) Patients (EDALINE)	LDE225 (Hedgehog inhibitor)	Docetaxel
NCT01596751	Metastatic Breast Cancer	PLX 3397 (multi-kinase inhibitor including CSF1R)	Eribulin
NCT01837095	Metastatic Breast Cancer	POL6326 (CXCR4 inhibitor)	Eribulin
NCT00754312	Newly DiagnosedBreast Cancer	SNDX-275 (Histone deacetylase inhibitor)	
NCT02154776	HR+, HER2-negative Post-menopausal Women With Advanced Breast Cancer	LEE011 (CDK4/6 inhibitor)	Buparlisib and Letrozole
NCT01467310	Triple Negative Breast Cancer	GSK1120212 (MEK1/2 allosteric inhibitor)	
NCT02158507	Metastatic Triple Negative Breast Cancer	Veliparib (ABT-888) (PARP inhibitor)	Lapatinib (Tykerb)

Abbreviations: HR, hormone receptor.

**Table 4:** Potential combinations for new agents in TNBC

Function	Family of compounds	Target/compound	Potential Combination	Development stage/rational
Cell division	Mitotic kinase inhibitors	Aurora kinase inhibitors PLK inhibitors TTK inhibitors	Chemotherapies. Taxane-based and platinum compounds	Clinical stage
Intracellular Signaling	PI3K/mTOR inhibitors	PI3K mTOR	Anti-androgen receptors	Overcoming mechanisms of resistance/Clinical stage
Intracellular Signaling	FGFR inhibitors	Nintedanib, ponatinib, dovitinib		Clinical stage
Intracellular Signaling	Erk5	TG02	Taxanes	Increase cell death
Intracellular Signaling	JAK/STAT	EC70124	Platinum compounds	Increase cell death/preclinical
DNA damage	PARP inhibitors	PARP Inhibitors: ABT-888, BSI-201 (Iniparib)	DNA damaging agents in BRCA mutated tumors	Increase cell death/ Clinical stage
DNA damage	ATR and Chek inhibitors	ATR Chek1 (LY2606368)	DNA damaging agents in ERCC1 and BRCA mutated tumors	Increase cell death/Clinical stage
Immunologic agents	Check point inhibitors	Anti-PD-1 antibodies: Pembrolizumab (MK-3475), Atezolizumab (MPDL3280A).	Taxanes	Clinical stage
Immunologic agents	ADCs	Sacituzumab Govitecan IMMU-132, Glembatumumab vedotin	Chemotherapy	Augment drug penetration/Clinical stage
Immunologic agents	Ab against macrophage colony-stimulating factor	MCS110	Carboplatin	Clinical stage
Targeting apoptosis	second mitochondrial-derived activator of caspases (SMAC) mimetic	LCL161	Taxanes	Clinical stage
Gene expression modulators	BET inhibitors	OTX015	Taxanes and DNA damaging agents	Induces cell cycle arrest/Clinical stage
Gene expression modulators	CDK7, CDK9 inhibitors	TG02, THZ1	Taxanes	Increase cell death
Gene expression modulators	CDK12 inhibitors	THZ1	PARP inhibitors	Preclinical stage
Gene expression modulators	HDCi	Romidepsin	DNA damaging agents (platinum-based)	Clinical stage

### Targeting the mitotic process

TNBC is characterized by its high proliferation rate [1, 11, 28]. Recent studies have demonstrated that kinases involved in the formation of the mitotic spindle, such as TTK/Mps1, polo-like kinases (PLKs) or NIMA/NEK2, are overexpressed in this particular breast cancer subtype [29]. Agents against TTK or PLKs have shown antitumor activity in preclinical models and are currently under clinical development [30–33]. In the case of NIMA, knockdown of this gene in triple negative tumors induced cell cycle arrest followed by cell death [34]. Similarly, targeting aurora kinases A and B has shown an antitumoral effect in TNBC and these agents were synergistic with MEK inhibitors and agents targeting microtubules [35, 36].

### Gene expression modulators

In a heterogeneous disease like TNBC targeting epigenomic components that can indirectly control several oncogenic functions represents an attractive approach. Bromodomain and extra terminal domain (BET) inhibitors and histone deacetylase inhibitors (HDACi) are two types of agents with an epigenetic mechanism of action that have preferentially been explored in TNBC. BET inhibitors belong to a new family of compounds that by inhibiting bromodomains can modify gene transcription [37]. These compounds have shown activity in several tumor types like neuroblastoma, where the expression of the transcription factor c-Myc was reduced; or in breast cancer by affecting key signaling elements involved in resistance to targeted agents [38, 39]. One of these compounds OTX015 is currently under early clinical development [40]. In preclinical models BET inhibitors have shown clinical activity in TNBC synergizing with chemotherapies used for the treatment of this disease [41, 42]. On the other side, HDACi have shown activity on TNBC tumor initiating cells [43, 44] and some of these agents are currently in phase II studies, mainly in combination with platinum-based therapies (see Table 2).

Through inhibition of the kinase activity of the cyclin-dependent kinases CDK7, CDK9 and CDK12 several agents have been reported to indirectly control transcription. These CDKs modulate the activity of the RNA polymerase II large subunit through phosphorylation at its C-terminal domain [45]. Such phosphorylation is required for initiation and elongation of transcription. A recent study has shown that CDK7 inhibition triggers cell death in preclinical models of TNBC, opening the door for the potential evaluation of CDK7 inhibitors in the clinical setting [46]. Moreover, the dual CDK7 and CDK9 inhibitory drug TG02 has shown antitumor effect in TNBC [47]. Interestingly, CDK12 has been associated with genomic stability [48], one of the characteristics of BRCA1/2 mutated TNBCs. In TNBC attenuation of CDK12 function increases the effect of PARP inhibitors [49].

As indicated above, the molecular classification of TNBC includes the luminal androgen receptor subtype, characterized by the expression of that receptor. In fact, approximately 10-15% of TNBCs express the AR [50, 51]. In this context several studies are exploring the role of targeting AR in this setting [52]. Single agent phase II studies have evaluated the clinical activity of anti-androgens in estrogen receptor negative or triple negative breast tumors. In these studies the clinical benefit rate defined as complete response, partial response and stable disease was around 40% at 4 months [53, 54]. Although the clinical activity of anti-androgens is not impressive, these data have stimulated the design of further studies in TNBC.

### Inhibiting tyrosine kinase receptors and intracellular signaling nodes

In addition to the targeting of molecules involved in nuclear processes such as those defined above, targeting cell surface or intracellular signaling molecules, mainly kinases, that may favor a prooncogenic phenotype has also been explored in TNBC. With respect to cell surface receptors, studies on the fibroblast growth factor receptor (FGFR) family have shown that 9% and 4% of TNBC have FGFR1 and FGFR2 amplification, respectively [55, 56]. Inhibition of these receptors has been shown to reduce tumor growth in preclinical models, supporting the evaluation of agents against FGFR in the clinical setting [57, 58].

In the case of signaling routes, particular emphasis has been placed in the PI3K/mTOR/AKT pathway. Molecular studies indicated that this route is activated in 10-15% of TNBC, and such activation may be caused by mutations, loss of suppressor phosphatases like PTEN or INPP4B; or in a small proportion of patients, amplification of AKT [59, 60]. Of note, PIK3CA mutations seem to be more frequent in the mesenchymal subtype [28]. Recent studies showed the relevant role of PI3K/mTOR inhibitors in TNBC and their synergistic interaction with chemotherapy [61]. Interestingly, inhibition of this route produced only a cytostatic effect, but when combined with chemotherapy the association induced cell death [61].

Another kinase with a relevant role in TNBC that has been recently described is ERK5. Expression of this protein was associated with worse outcome; and agents inhibiting its function produced an anti-proliferative effect [47, 62]. Combination of ERK5 kinase inhibitors with chemotherapy was synergistic [47].

Targeting multiple key intracellular signaling nodes can be a better strategy than inhibiting single kinases [63]. In line with that idea novel multikinase inhibitors have shown preclinical activity mainly when targeting the PI3K/mTOR and the JAK/STAT pathways [64].

Finally, it should be mentioned that strategies aimed at inhibiting EGFR did not meet expectations. Clinical studies showed no signs of potential activity when combined with chemotherapy [65]. A similar lack of clear efficacy was observed for antiangiogenic therapies, with studies showing no benefit [66]. Even considering the lack of data to support these strategies in the clinical setting, some studies are currently exploring novel compounds against these targets in clinical trials, as can be seen in Table 2.

### Immunologic agents: check point inhibitors and antibody drug conjugates (ADCs)

Strategies based on modulating antitumoral immune responses have demonstrated clinical efficacy in different oncological diseases. Among the various strategies tested, those using antibodies aimed at augmenting the cellular immune response against tumors, particularly with the use of checkpoint immunologics, and agents directed to cell surface proteins have progressed to the clinic.

With respect to agents aimed at modulating the cellular immune response, blockade of cytotoxic T-lymphocyte antigen-4 (CTLA-4) and programmed cell death protein-1 (PD-1) or its ligand (PD-L1) with antibodies has shown clinical benefit in different indications [67]. In breast cancer, expression of PD-L1 has been described in 20% of TNBC [68]. Breast tumors with presence of CTLA-4 have been linked with a worse outcome [69]. However, expression of these antigens cannot fully predict response to antibodies targeting them. A recent clinical study with the anti-PD-1 antibody pembroluzimab has shown evidence of clinical activity with a good safety profile [70]. In this context, some phase III studies are currently analyzing the antitumoral action of anti-PD-1 antibodies either as monotherapy or in combination with chemotherapy (see Table 1).

In addition to those strategies that have shown potential clinical efficacy, the immunomodulatory properties of the colony stimulating factor 1 (CSF-1) could be manipulated for treating TNBC. Knocking out CSF-1 in mice susceptible to breast cancer resulted in reduced macrophage recruitment to the tumor and delayed metastasis [71]. This may be a consequence of the immunosuppressive action of tumor-associated macrophages. In the clinical setting the ratio among neutrophils and lymphocytes predicts worse outcome when it is higher than a certain level, also suggesting the deleterious role of neutrophils on cancer [72]. In preclinical models the role of neutrophils in relation to lung colonization of metastases in breast cancer has recently been reported [73]. CSF-1-receptor inhibition increased CD8+ T cell tumor infiltration and enhanced antitumor response to paclitaxel in xenograft models [74]. Agents with this mechanism of action are currently in phase II studies (Table 2).

The second group of immunologic agents is based on the recognition of cell surface proteins by antibodies, or modified versions of antibodies carrying a cytotoxic drug. Two novel ADCs are in clinical development. IMMU-132 is an ADC against Trop-2 conjugated to the irinotecan metabolite SN-38 [75]. Trop-2 is a calcium signal transducer transmembrane protein that is expressed in normal tissue and overexpressed in many tumors [76]. IMMU-132 has shown promising signs of activity in early clinical studies [77]. IMMU-132 has received fast track designation from the FDA for metastatic TNBC (Table 1). Glembatumumab vedotin is an ADC against the glycoprotein non-metastatic B (gpNMB) expressed on the membrane of cancer cells. gpNMB was associated with worse outcome in breast cancer patients, particularly those with a triple negative phenotype [78]. In this case the antibody is linked to monomethyl auristatin E, a potent antimitotic agent [78]. Glembatumumab vedotin has demonstrated promising signs of activity in TNBCs overexpressing gpNMB and is now in clinical evaluation [79]. Other ADCs include those against the folate receptor (see Table 2).

### Pathways involved in stemness

TNBC is a subtype of cancer where subpopulations of cells with stem-like characteristics are present. In this context, targeting signaling pathways involved in biological functions associated with the maintenance of the stem cell phenotype, has been explored preclinically. The JAK/STAT pathway is preferentially active in basal-like tumors [64, 80]. Inhibition of this pathway with kinase inhibitors produces tumor regression in preclinical models [64, 80]. Another pathway that participates in the maintenance of stem cell properties is the Notch signaling pathway [81]. Mutations at NOTCH1, NOTCH2 and NOTCH3 and rearrangement at NOTCH1 have been described, and inhibition of NOTCH cleavage with gamma-secretase inhibitors has shown activity in preclinical models [82, 83]. Some studies are currently evaluating this family of compounds in TNBC (see Tables 2 and 3).

### Inducing cell death by targeting apoptosis

Direct induction of cell death by using agonists of cell death receptors or inhibitors of anti-apoptotic proteins can represent another antitumoral strategy in TNBC. Agents that act on inhibitors of apoptotic proteins (IAPs) have shown potential signs of activity in different preclinical models [84, 85]. LCL-161 is a second mitochondrial-derived activator of caspases (SMAC) mimetic that has shown clinical activity in TNBC in combination with paclitaxel in the neoadjuvant setting, when selecting patients with a specific gene signature [86]. Recently, two articles have described the role of PIM kinase inhibition as an effective antitumor strategy in basal-like tumors [87, 88].

## OPTIMIZING COMBINATORIAL STRATEGIES

Given the fact that TNBC is a heterogeneous disease, combined inhibition of relevant vulnerabilities seems to be a better approach than targeting individual events. This is in line with the clinical experience which demonstrated that targeting of individual routes often results in escape of inhibition of tumor growth by secondary activation of other protumorigenic routes. The development of novel drug combinations to improve management of patients with TNBC requires identification of tumors that most likely would respond to a given combination. In addition, and given the fact that TNBCs are sensitive to classical chemotherapies, the new drug is expected to be tested in combination with one of those chemotherapies. Below we will discuss drug combination strategies focusing on opportunities for clinical development and their limitations.

### DNA-damaging agents

The finding that a number of TNBC present alterations in DNA repair mechanisms opened the possibility of exploiting this situation and fostered the development of PARP inhibitors. PARP inhibitors are synergistic with agents that induce double strand breaks. Among chemotherapies that produce double strand breaks, platinum compounds and temozolomide are the most used in the clinical setting, for glioblastoma and TNBC, respectively (Tables 2 and 3).

The adequate use of PARP inhibitors in combination with other compounds provoking DNA damage relies on the identification of tumors that may be sensitive to the combination. In this respect, mutations in BRCA1 and BRCA2 genes have been reported to be the principal predictors of response to PARP inhibitors. Similarly, molecular alterations in other DNA-repair genes, including ATM, Fanconi's anemia genes or CHEK2, predict response to PARP inhibitors in prostate cancer [89].

The principal mechanism of resistance to PARP inhibitors includes the presence of mutations at the BRCA1/2 genes that restore their function [90, 91]. The presence of alternative splicing of the BRCA gene has also been suggested as a potential mechanism of resistance [92].

### BET and CDK inhibitors

Association of BET inhibitors has shown to be synergistic with chemotherapy in preclinical models of lymphoma and TNBC [37, 42, 93]. Recent data in hematological malignancies suggest that WNT signaling and rewiring of transcriptional programs are potentially associated with primary and secondary resistance to BET inhibitors [94]. Preclinical data suggest that inhibition of CDK12 can increase the effect of PARP inhibitors [49]. The CDK7/9 inhibitor TG02 was shown to synergize with chemotherapy, especially with taxotere, in TNBC cellular models [47].

### Kinase inhibitors

As mentioned before, a wide range of these agents have been evaluated in combination. The reason behind this is the complementary antitumoral action when combining several kinase inhibitors. In addition, such combinations may be more effective by inhibiting mechanisms of resistance that were developed by activation of secondary loops [95]. In this context the main limitation for targeted combinations, beyond the lack of efficacy in the clinical setting, is related to the overlapping of toxicities, mainly when different kinase inhibitors are combined. For example, preclinical data suggested the synergistic antitumoral effect when combining PI3K and MEK inhibitors [96, 97]. However early phase studies evaluating PI3K and MEK inhibitors in combination showed poor tolerability [98]. The combinations of drugs targeting both pathways seemed to have greater activity in KRAS mutated tumors, mainly ovarian tumors, but the high grade of toxicity observed, limited the clinical development [98].

An alternative approach for the clinical development of these compounds could be to combine these agents with standard chemotherapy. This is for instance the case for the association of PI3K inhibitors with taxanes. While targeting the PI3K/AKT/mTOR pathway by itself is only cytostatic, combination of PI3K inhibitors with agents targeting mitosis produces a cytotoxic effect in preclinical models [61]. That circumstance could translate into an increase in efficacy in the clinical setting. In fact, such strategy is now being evaluated in some clinical studies (Tables 2 and 3). A similar approach has been taken for the clinical development of spindle checkpoint kinase inhibitors that are under evaluation in combination with taxanes (Tables 2 and 3). Finally, combinations of PI3K inhibitors with anti-androgens or PARP inhibitors are under evaluation, as described in other parts of this review.

### Immunologic agents

Predicting which patients may respond to an immune-based antitumoral strategy remains one of the aspects that still requires much research. In the case of ADCs, the first step in their use is to define the degree of expression of the target that would confer therapeutic advantage. In this respect, preclinical data obtained in other tumors indicates that overexpression of the target is usually accompanied with sensitivity. The use of novel techniques like positron emission tomography imaging of radioactively labeled antibodies (immunoPET) could help selecting those patients that will have a clinical response [99].

One of the potential mechanism of resistance to ADCs, as happens with antibodies, is associated with the limited drug distribution within the tumor due to their high molecular weight or tumor vascularization [100]. Similarly, elimination of chemotherapy from the intracellular compartment to the extracellular matrix could be another mechanism of resistance [101]. In this context, it has been suggested that chemotherapy can augment the activity of ADCs by destroying tumoral tissue, therefore facilitating the penetration of ADCs in different tumoral areas [102]. In the same line, new ADCs are designed to increase the payload ratio of chemotherapy linked with each antibody [103], so the amount of drug that reaches the tumor is higher. Another attractive possibility could be the use of ADCs targeting the stroma in combination with antitumor ADCs.

How to boost the anticancer properties of immune checkpoint modulators is an intensive area of research. In this case, the presence of various immune modulators in the tumor microenvironment appears to have an important role. Expression of checkpoint regulators PD-1, PD-L1 or CTLA-4 appears to be necessary but not sufficient to explain sensitivity to blocking antibodies against them. Some other cellular components of the tumor stroma, such as tumor-associated macrophages, myeloid-derived suppressor cells, or Treg lymphocytes may create an immunosuppressive environment that could prevent a strong antitumoral response by cytotoxic T lymphocytes. Some studies have suggested that the kinase inhibitor ibrutinib, that inhibits the BTK and ITK kinases, when added together with PD-L1 inhibitors, can boost the immune response by modulating the balance among T lymphocytes [104]. In several tumor types, combination of PD-L1 inhibitors with anti-VEGF strategies is under clinical evaluation [105]. However, combinations should be explored with caution: for instance the association of vemurafenib to ipilimumab in melanoma showed an unacceptable hepatotoxicity limiting therefore their clinical development [106].

### PI3K and AR inhibitors

Given the fact that the LAR subtype is the one more enriched with mutations at the PIK3CA gene [28], studies evaluating the combination of anti-androgens with PI3K inhibitors are currently ongoing.

## CONCLUSIONS

A wide range of targeted agents against different oncogenic and non-oncogenic vulnerabilities are currently under evaluation in TNBC. Unfortunately, classification of breast cancer by transcriptomic profiles has not contributed substantially to the identification of new targets or to the stratification of patients for new compounds. Indeed, the role of these subtypes in the clonal evolution of cancer tumors is under evaluation at this moment, with preliminary results not supporting a major role [107].

PARP inhibitors are currently in the late stages of drug development in TNBC patients with mutations at BRCA genes, suggesting that these compounds could be incorporated to the therapeutic armamentarium to fight this disease in the near future. Other families of agents with promising activity are novel ADCs and immune checkpoint inhibitors, both with ongoing phase III studies. On the other hand, the major limitation for all these compounds is the identification of biomarkers of response or companion diagnostics.

How to integrate these agents for treatment combinations is challenging and should always try to avoid the overlapping of toxicities. A proper evaluation of the selected combination in preclinical models should be mandatory before clinical testing. Finally, given the fact that chemotherapy is the standard of care in TNBC, the development of novel combinations will probably contemplate the use of the compounds together with a chemotherapeutic agent.
